# Safety and Outcomes of Colonoscopy in Cardiac Transplant Candidates with Severely Versus Non-severely Reduced Left Ventricular Function: A Single-Center Retrospective Cohort

**DOI:** 10.1007/s10620-025-09371-7

**Published:** 2025-09-17

**Authors:** Noor Hassan, Mir Zulqarnain, Chase Branstetter, Ifrah Fatima, Islam Mohamed, Vinay Jahagirdar, Jagadish Koyi, Saqr Alsakarneh, Ameen Awad, Misha Gautam, Abbas Bader, Sruthi Sripada, Mohamed Ahmed, Adel Muhanna, Thomas Cunningham, Wendell Clarkston

**Affiliations:** 1 Internal Medicine, University of Missouri Kansas City School of Medicine, Kansas City, MO USA; 2 Saint Luke’s Health System, Kansas City, MO USA

**Keywords:** Colonoscopy, Cardiac transplantation, Colorectal cancer

## Abstract

**Background:**

Pre-cardiac transplant evaluation, including colorectal cancer screening, is vital for organ allocation. Data on colonoscopy risks in transplant candidates are limited to retrospective analyses with varying methodologies. Literature assessing colonoscopy alone without esophagogastroduodenoscopy is even more limited. We studied adverse events and clinical outcomes of pre-transplant screening colonoscopy in patients stratified by ejection fraction (EF).

**Methods:**

Charts were reviewed at Saint Luke’s Hospital in Kansas City, Missouri, between 2014 and 2023. Cohorts were divided by EF: severe (EF < 30%) and non-severe (EF ≥ 30%). Demographics and clinical outcomes were compared using descriptive statistics and chi-square tests, with a *p*-value: < 0.05. Outcomes included adverse events and adenoma or colorectal cancer detection.

**Results:**

Among 322 patients, 231 had EF < 30% and 91 had EF ≥ 30%. Adverse events were similar in both cohorts (*p* > 0.05 for all). No severe events were observed during colonoscopy as classified by the American Society of Gastrointestinal Endoscopy (Cotton et al. (2010) Gastrointest Endosc. 71:446–454) Adenoma detection rate in the EF ≥ 30% group was 37.1 and 26.6% in the EF < 30% group. Analysis showed no significant differences in adenoma detection (*p* = 0.08).

**Conclusions:**

Our study provides new data on the safety and diagnostic performance of pre-transplant colonoscopy in patients with severe and non-severely reduced EF. While minor adverse events were commonly observed, no severe complications occurred during colonoscopy. Adverse clinical outcomes were comparable between cohorts regardless of EF severity, and severity did not appear to significantly impact detection of adenomas; however, bowel prep and colonoscopy were performed in a closely monitored, inpatient setting.

## Background

With an aging global population and rising burden of cardiovascular disease, the use of advanced therapeutic modalities in chronic disease management has become increasingly important. Given this growing need, safety evaluation of innovative therapies remains essential to guide their effective utilization and optimize clinical outcomes. Cardiac transplant is known to be the optimal treatment for irreversible and severely advanced heart failure (HF) [1, 2]. The prevalence of American adults with HF is expected to increase by approximately 2 million by 2030 [3]. Considering the shortage of donor organs, stringent pre-transplant evaluation criteria will remain necessary.

One critical consideration in the pretransplant evaluation process is the presence of malignancies. Pre-transplant malignancy is linked to decreased survival, and active malignancy, other than melanoma, is a contraindication for transplantation [4]. Various factors have been proposed to explain this increased risk. Perhaps the most cited explanation is the potential role of post-transplant immunosuppressive medications. Christenson et al. found that colorectal cancer (CRC) in solid organ transplant recipients is associated with a higher incidence of PIK3CA mutations and mismatch repair-deficient (MMRd) disease, highlighting a link between immunosuppressants and CRC [5]. The prevalence of malignancy among cardiac transplant candidates has previously been noted as high as 10%, with CRC being one of the most common [6].

Colonoscopy is the primary modality for diagnosing and treating various colorectal conditions, including advanced polyps and cancer. Complications from colonoscopy in average-risk individuals are rare, but they remain a concern in patients with complex comorbidities. Heart failure increases the risk of adverse events in colonoscopy through several mechanisms.

First, reduced cardiac output and limitations of cardiac reserve predispose patients to hypotension, cardiac arrhythmias, and other cardiovascular complications during sedation and procedure [7]. Second, adult patients undergoing pretransplant evaluation often present with additional comorbidities such as chronic kidney disease, diabetes mellitus, obesity, atrial fibrillation, and chronic respiratory disease which further exacerbate cardiopulmonary, infection, and bleeding risks [8]. Third, patients are often on medications such as antiplatelet and anticoagulant agents. Fourth, frailty and decreased functional status are often seen in patients with end-stage heart failure and increase susceptibility to adverse outcomes though mechanisms such as malnutrition, chronic inflammation, and other neural and hormonal dysregulation [7, 9].

The increased risk of colonoscopy in patients with heart failure has been previously documented and is especially notable in acute exacerbation [10]. There is, however, a notable lack of dedicated safety data available on screening colonoscopy in patients undergoing routine cardiac transplant evaluation. This study aims to evaluate the risks and clinical outcomes specific to colonoscopy for pre-cardiac transplant screening in patients with end-stage heart failure.

## Methods

### Study Design

We completed a retrospective cohort study of patients undergoing colonoscopy as part of their pre-cardiac transplant evaluation. This study was conducted at a single tertiary transplant center using data from charts between April 2014 and August 2023 at Saint Luke’s Hospital System in Kansas City. All patients that underwent pre-cardiac transplant screening colonoscopy during this period were included. All patients were admitted to the hospital for expedited multidisciplinary transplant workup as part of the system’s standard transplant evaluation protocol and monitored closely by a team of cardiologists, subspecialists, anesthesiologists, nursing, and other staff. Notably, patients were evaluated by advanced HF and transplant-trained cardiologists that gave approval before procedures such as endoscopy were performed. Endoscopy was performed by gastroenterologists with greater than 5 to 10 years of experience; the majority of endoscopists had greater than 10 years of experience. Colonoscopies were scheduled for 45-min time slots. St. Luke’s exclusively utilizes the Fujinon endoscope, and scopes were upgraded every 5 to 6 years to keep up with current models. Bowel preparation was most commonly a GoLytely split prep; however, Moviprep was also utilized. Patients were started on a clear liquid diet the day before the procedure. As part of the system’s protocol, all patients undergoing cardiac transplant received a colonoscopy as part of their evaluation if they were not up to date on screening colonoscopy. The study was undertaken in alignment with national ethics guidelines; patient demographics and clinical outcomes data were abstracted from the electronic medical record (EMR) after Institutional Review Board approval. Given the retrospective study framework, data were deidentified before publication and informed consent was exempted.

Data included quality and safety measures such as adverse events and effects. Outcome measures included rate of detection for adenoma, high-grade dysplasia, and colorectal malignancy prevalence. Medications around the procedure were also tracked including anticoagulants, antiplatelets, infusions, inotropes, and sedation medications. Significant adverse events were initially assessed by severity based on the lexicon recommended by the American Society of Gastrointestinal Endoscopy workshop [11]. However, more minor adverse events without serious clinical consequences were recorded when no severe-range adverse events occurred during colonoscopy. Key outcomes were assessed through chart review. Data were collected primarily by internal medicine resident physicians. Disagreements and data concerns were resolved by the senior gastroenterology fellow and the supervising gastroenterology physician and principal investigator. All data collectors utilized a protocol-driven data abstraction tool based on existing literature. All patients in the study underwent colonoscopy in the hospital setting as part of their transplant protocol.

### Study Sample

The study had a sample size of 322 participants. Patients were assigned to two cohorts based on ejection fraction (EF): severe (EF < 30%) and non-severe (EF ≥ 30%) during the time of colonoscopy. The EF cutoff of 30% was chosen based on literature classifying an EF < 30% as severely reduced [12, 13]. With regards to safety risk, more robust literature is available describing patients with heart failure undergoing noncardiac surgery including those with reduced EF [7]. Retrospective studies in heart failure patients undergoing colonoscopy have shown increased risk of adverse events in patients with EF < 30% but have significantly lower power in comparison [10].

### Statistical Analysis

Data were obtained from two EMR systems (EPIC and gGastro). We conducted descriptive statistics, including chi-square tests, to compare the outcomes between the two groups. Patient characteristics were summarized with mean and range, or number and percent, and compared between those with and without abnormalities by the Wilcoxon rank sum test or the Fisher’s exact test as was appropriate. Study data were collected and managed using REDCap electronic data capture tools hosted at Saint Luke’s Hospital System Kansas City [14, 15]. Data analysis was performed using Microsoft Excel (versions 2013–2019) for Windows (Microsoft Corp., Redmond, WA, USA).

## Results

The number of patients that underwent pre-transplant screening colonoscopy over the study period was *n* = 322. The participants were divided into two cohorts by severity of EF.

In the severely reduced EF cohort (< 30%) there were 231 patients, 17.7% were female, and the average age was 56 years. Pertinent medical history included arrhythmia (primarily atrial fibrillation) at 63.2% with documented history, 36.4% had obstructive sleep apnea (OSA), 19.9% had chronic obstructive pulmonary disease (COPD) or asthma, and 16.9% had pulmonary hypertension (pHTN). Other demographic data were also included; see Table 1. In the non-severely reduced EF cohort (≥ 30%) there were 91 patients, slightly more were female at 25.2%, and the average age was younger at 43.5 years. Previous medical history included arrhythmia (also primarily atrial fibrillation) but at a lower prevalence with 49.5% documented history, more patients in this cohort had OSA (47.3%), a similar percentage of patients had COPD or asthma (23%), and the same percentage of 18.7% was seen for pHTN.

**Table 1:** Baseline characteristics

Characteristics	Severe EF < 30%	Non-severe EF ≥ 30%
Participants	*n* = 231	*n* = 91
Male	190 (82.3%)	68 (74.8%)
Female	41 (17.7%)	23 (25.2%)
Hx of Arrhythmia	146 (63.2%)	45 (49.5%)
OSA	84 (36.4%)	43 (47.3%)
Pulmonary HTN	39 (16.9%)	16 (18.7%)
COPD or Asthma	46 (19.9%)	21 (23%)
Mean Age (years)	56	43.5

### Anticoagulant and Antiplatelet Use

Use of antiplatelet (aPLT) and anticoagulant (AC) therapy was also recorded. In the severe EF cohort, the percentage of patients on either of these medications was 72.7%. No patients in this cohort were on triple therapy. The percentage of individuals on both aPLT and AC was 19%. Those on AC alone represented 13%. Patients on dual aPLT (DAPT) made up 9%. Patients on aPLT monotherapy made up 33% of the severe EF cohort.

In the non-severe EF cohort, use of either aPLT or AC was comparatively higher at 82.4%. There was one patient on triple therapy. The percentage of individuals on both aPLT and AC was lower at 14.1%. Those on AC alone were similar in both cohorts at 13%. There was a higher percentage of patients on DAPT (12%). Similarly, a higher percentage of patients were on antiplatelet monotherapy (42.2%) in the non-severe cohort (Table 2).

**Table 2:** Anticoagulant and antiplatelet regimen

AC or aPLT Agent	Severe EF < 30%	Non-Severe EF ≥ 30%
On either	168 (72.7%)	75 (82.4%)
Triple Therapy	0 (0%)	1 (1.1%)
AC + aPLT	43 (18.7%)	13 (14.1%)
AC only	29 (12.6%)	12 (13.0%)
DAPT	20 (8.7%)	11 (12.0%)
aPLT only	76 (33%)	38 (42.2%)

### Sedation Methods

Agents used for sedation were also recorded between cohorts. Almost everyone in both the severe EF and non-severe EF cohorts received propofol-based sedation with 97 and 98.9%, respectively. In the severe EF cohort, seven patients did not receive sedation with propofol (3%). In the non-severe EF cohort, the sedation utilized was even more homogenous, with 1 patient who did not receive propofol-based sedation. Other agents that were used included Etomidate, Ketamine, Fentanyl, and Versed (Table 3).

**Table 3:** Sedation method

Sedation agent	Severe EF *n* = 231	Non-Severe EF *n* = 91
**Propofol based:**	224 (97%)	90 (98.9%)
Propofol only	122 (52.8%)	56 (61.5%)
Propofol + Etomidate	34 (14.7%)	8 (8.8%)
Propofol + Fentanyl	30 (13.4%)	10 (11.0%)
Propofol + Fentanyl + Ketamine	7 (3%)	1 (1.1%)
Propofol + Etomidate + Ketamine	7 (3%)	4 (4.4%)
Propofol + Ketamine	6 (2.6%)	6 (6.7%)
Propofol + Versed	6 (2.6%)	4 (4.4%)
Propofol + Fentanyl + Versed	5 (2.2%)	1 (1.0%)
Propofol + Fentanyl + Etomidate	2 (0.9%)	0 (0%)
Propofol + Fentanyl + Etomidate + Ketamine	2 (0.9%)	0 (0%)
Propofol + Ketamine + Versed	2 (0.9%)	0 (0%)
Propofol + Etomidate + Versed	1 (0.4%)	0 (0%)
**Without Propofol:**	7 (3%)	1 (1.1%)
Etomidate only	3 (1.3%)	0 (0%)
Etomidate + Fentanyl	1 (0.4%)	0 (0%)
Etomidate + Ketamine	1 (0.4%)	0 (0%)
Etomidate + Ketamine + Versed	1 (0.4%)	0 (0%)
Fentanyl + Ketamine	1 (0.4%)	0 (0%)
Fentanyl + Versed	0 (0%)	1 (1.1%)

### Adverse Events

No adverse outcomes during colonoscopy met the criteria for definition via the ASGE Lexicon greater than mild or moderate classification. As such, the definition for adverse events was expanded to include minor events to add data for quantitative assessment. Comprehensive analysis of secondary adverse clinical outcomes was performed including bleeding, arrhythmia, hypotension, and hypoxia. No statistically significant differences were observed in adverse clinical outcomes between the EF ≥ 30 and EF < 30 groups (*p* > 0.05 for all).

In total between both cohorts, there were no cases of perforation; 2 cases of bleeding occurred, 114 experienced hypotension, 27 sustained arrhythmias, and hypoxia occurred in 21 participants. Six episodes of hypoxia were noted in the EF ≥ 30% group (6.6%) and 15 episodes (6.6%) were noted in the EF < 30%. Hypoxia was defined as requiring > 2 L of oxygen during the procedure to titrate SpO2 to > 92%. Hypoxic events were corrected by routine supplemental oxygen and did not require abortion of endoscopy, endotracheal intubation, or ICU admission. The definition of arrhythmia included atrial fibrillation, non-sustained ventricular tachycardia, high burden of premature ventricular contractions (PVCs), bradyarrhythmias, and other supraventricular arrhythmias. No episodes of ventricular fibrillation or other serious arrhythmia requiring Advanced Cardiac Life Support (ACLS) intervention or abortion of procedure were sustained (Table 4).

**Table 4:** Adverse events

Adverse event	Severe EF *n* = 231	Non-severe EF *n* = 91	P-value
Hypotension	84 (36.4%)	30 (33%)	0.57
Hypoxia	15 (6.6%)	6 (6.6%)	0.98
Arrhythmia	21 (9.1%)	6 (6.6%)	0.48
Bleeding	1 (0.4%)	1 (1.1%)	0.49
Angioectasias	1	1	–
Perforation	0	0	–
Death	0	0	–
Cardiac Arrest	0	0	–
ICU Admission	0	0	–
Emergent Intubation	0	0	–
Aborted Procedure	0	0	–
Hospitalization in 30 days	2	0	–

Repeat hospitalization rates within 30 days were also monitored. We found that 2 out of the 322 patients (< 1%) were hospitalized within the period. Both were in the severely reduced EF cohort. The indication of hospitalization for the first patient was right ventricular dysfunction sustained after left ventricular assist device placement (LVAD). The second was for a heart failure exacerbation 11 days after colonoscopy leading to cardiorenal syndrome and renal failure.

### Colorectal Cancer Screening Results

In the EF ≥ 30 group the adenoma detection rate was 37.1% of cases, with high-grade lesions detected in 1.8%, and no findings of CRC. In the EF < 30 group there was an adenoma detection rate of 26.6%, high-grade lesions in 1.1%, and CRC in 1.1%. According to Rex et al., the task force recommended a minimum target for an overall ADR of 25% in mixed gender screenings during the time period in which data were collected for the study [16]. The reported number of participants with poor bowel preparation was nearly identical between the severe EF (6.5%) and non-severe EF (6.6%) cohorts (Table 5).

**Table 5:** Detection rates and prep quality

Finding	Severe EF (*n* = 231)	Non-Severe EF (*n* = 91)	P-value
Adenoma Detection Rate	26.6%	37.1%	0.08
High-Grade Lesions	1.1%	1.8%	–
Colorectal Cancer	1.1%	0.0%	–
Poor Prep	6.5%	6.6%	–

## Discussion

Organ transplant recipients face a significantly elevated risk of colorectal cancer (CRC) and have higher mortality following diagnosis compared with the general population [17]. Detecting and removing any adenomas before transplantation is therefore a critical component of pre-transplant care.

Existing data on the safety of colonoscopy in heart transplant candidates are sparse and derived mostly from small, retrospective studies. For example, Ehlken et al. reported an unfavorable risk–benefit profile for gastrointestinal endoscopy screening prior to heart or lung transplant, though nearly all participants underwent EGD and only seven received colonoscopy alone; nevertheless, serious adverse events, including a death (cardiac electromechanical dissociation) and a cecal perforation, were observed during colonoscopy [18]. Similarly, Abu et al. noted increased complications and mortality among patients with severe HF undergoing endoscopy, though many presented in acute decompensation and again a large proportion of analyses included EGD [10]. These limitations make it difficult to draw conclusions about the specific risk profile of colonoscopy in advanced HF populations.

In our single-center retrospective study of 322 cardiac transplant candidates, no severe colonoscopy-related adverse events were observed in either the severe or non-severe EF cohorts. Minor adverse events—including hypoxia, arrhythmia, and transient hypotension—were common, but there were no significant differences between EF cohorts (*p* > 0.05 for all comparisons). Importantly, all procedures were performed in the inpatient setting with continuous monitoring and multidisciplinary involvement, including advanced HF-trained cardiologists who optimized procedural timing. While our study was not powered to adequately detect rare but serious complications, these findings provide supportive—but certainly not definitive—evidence for the safety of colonoscopy as part of the pre-transplant evaluation in patients with advanced heart failure.

Patients with advanced HF remain a high-risk population for endoscopy. Optimal practice should include comprehensive cardiovascular assessment before colonoscopy, pre-procedural optimization of medications and comorbidities, and careful management of fluid status. Close collaboration with experienced anesthesiologists is critical to mitigate cardiopulmonary instability. Diligent post-procedural monitoring is also warranted to promptly identify delayed complications.

In patients with advanced cardiopulmonary disease who face elevated procedural and sedation risk, capsule endoscopy, CT colonography, and stool DNA-FIT testing represent reasonable alternatives [19, 20]. Second-generation capsule endoscopy has excellent sensitivity for polyps ≥ 10 mm (95–97%) but reduced sensitivity for polyps ≥ 6 mm (79–96%), with completion rates ranging from 57 to 92% depending on bowel preparation quality [21, 22]. CT colonography provides 67–94% reported sensitivity for adenomas ≥ 10 mm but performs poorly for smaller or flat lesions [23]. DNA–FIT testing avoids sedation and bowel prep with 92% reported sensitivity for CRC detection but only 42% sensitivity for advanced adenomas [19], and positive results still require colonoscopy. While these alternatives reduce procedural risk, they may lack the diagnostic precision—particularly for smaller lesions—needed in higher-risk transplant candidates, where maximizing cancer and adenoma detection is critical. Colonoscopy remains the gold standard in average-risk cohorts, offering the highest sensitivity and specificity for advanced adenomas and CRC, while also permitting biopsy, therapeutic intervention, and direct visualization [19, 23, 24]. Consequently, colonoscopy continues to be employed for routine screening in the Saint Luke’s Hospital transplant program, balancing its diagnostic superiority against the elevated procedural risks in advanced heart failure.

Future research in this area would benefit from larger multicenter retrospective and prospective studies to better characterize the factors influencing the safety and effectiveness of colonoscopy in patients with advanced heart failure and cardiac transplant candidates. Areas of particular interest include the impact of different sedation protocols, the role of pre-procedural optimization strategies, and the long-term outcomes of patients undergoing colonoscopy as part of pre- and post-transplant evaluations. Additional work is also warranted to examine markers of cardiac function beyond left ventricular ejection fraction, such as right ventricular function and pulmonary pressures. Finally, prospective studies directly comparing the diagnostic yield, safety, and patient-centered outcomes of colonoscopy with alternative CRC screening strategies—such as DNA–FIT with follow-up colonoscopy for positive results, CT colonography, and capsule endoscopy—would provide valuable evidence to inform clinical decision-making in this high-risk population.

## Limitations

Small, single-center retrospective studies are inherently limited in their ability to report on safety measures for several reasons, including inability to control confounders, lack of power to capture rare events, vulnerability to inconsistent or selective documentation, and variable generalizability to populations outside of the home institution. This was a retrospective study subject to these flaws and imperfections.

Given the limited number of cases in our dataset (322 patients) and the relative safety of colonoscopy procedures resulting in few clinically significant complications, we encountered challenges in conducting regression analyses. The scarcity of complications made it impractical to establish correlations with baseline characteristics. As a result, multivariate analyses were not calculated, which was a significant limitation in the study.

Consequently, we chose an alternative approach. We initiated our analysis with a baseline measure, in this case, the EF, and correlated it with the observed outcomes. We then compared the clinical outcomes between these two groups. Baseline characteristics assessed between cohorts had similarities, but the most notable exception was a difference in age. The mean ages for the severe EF and non-severe EF cohorts were 56 and 43.5 years, respectively. This was a major limitation in our choice of EF as the baseline measure to stratify cohorts.

Although we employed statistical methods to help adjust for simple known confounders, there are three notable variables missing from our data—right ventricular function, pulmonary pressures, and functional capacity. These are important clinically significant risk factors that were not available in our data set. It is likely that these major variables play some role in the observed age difference in our cohorts, and their omission, combined with the age gap, represents one of the greatest limitations of the study.

Additional limitations included lack of data collection on racial differences and the education level of participants. There is, however, State-level data from Kansas and Missouri available for demographic data. Epidemiologic studies on heart failure patients in these states—and other Midwest states—tend to be older and with a higher proportion of non-Hispanic White individuals; however, there is a significant representation of African American patients—especially in urban centers such as Kansas City [3]. In general, data from the United Network for Organ Sharing (UNOS) and the Scientific Registry of Transplant Recipients (SRTR) show that the demographics for adult heart transplant recipients are more commonly non-Hispanic White males, with a median age in their 50’s, but with some African American and Hispanic patients represented in these reports [3, 25]. It is suspected that the patient population studied in our cohorts reflects these broader statistics, but it cannot be confirmed.

## Conclusion

Our study provides data on the safety profiles of pre-transplant colonoscopy by comparing patients with severe (EF < 30%) and non-severe left ventricular ejection fraction (EF ≥ 30%) and tracking their adenoma and colorectal cancer detection rates. Given the limitations of this single-center retrospective study and the modest sample size, our findings should be interpreted cautiously and viewed as hypothesis-generating. In this study, the incidence of adverse clinical outcomes did not differ between patients with severe and non-severe EF. In addition to this, EF severity did not appear to significantly impair the effectiveness of colonoscopy in detecting colorectal neoplasms. Transient hypotension, hypoxia, and arrhythmia were common adverse events associated with pre-transplant colonoscopy in both HF cohorts. Given these findings, and rare but serious adverse outcomes noted in previous literature, colonoscopy should be performed after thorough cardiovascular risk stratification and only in centers that routinely manage the specific challenges inherent to high-risk cardiopulmonary patients. Potential areas for future research were also identified, focusing on opportunities to study interventions that could modify the risk profile of cardiac transplant candidates undergoing colorectal cancer screening.

## Data Availability

The dataset analyzed during this study is available from the corresponding author upon request.

## Abbreviations

CRC: Colorectal Cancer
HF: Heart Failure
EF: Ejection Fraction
OSA: Obstructive Sleep Apnea
pHTN: Pulmonary Hypertension
COPD: Chronic Obstructive Pulmonary Disease
DAPT: Dual Antiplatelet Therapy
AC: Anticoagulation
LVAD: Left Ventricular Assist Device
CVA: Cerebrovascular Accident
MI: Myocardial Infarction
MMRd: Mutations and Mismatch Repair-Deficient Disease
ADR: Adenoma Detection Rate
Hx: History
FIT: Fecal Immunochemical Test
